# Zufallsbefund von Lymphangioleiomyomatose in gynäkologischen Operationspräparaten – eine Fallserie

**DOI:** 10.1007/s00292-025-01414-0

**Published:** 2025-02-07

**Authors:** Julia Müller, Blake Gilks, Jessica McAlpine, Grit Gesine Ruth Hiller, Anne Kathrin Höhn, Lars-Christian Horn

**Affiliations:** 1 Arbeitsgruppe Mamma‑, Gynäko‑, Perinatalpathologie, Institut für Pathologie, Universitätsklinikum Leipzig AöR, Liebigstr. 26, 04103 Leipzig, Deutschland; 2 Department of Anatomical Pathology, Vancouver General Hospital and the University of British Columbia, Vancouver, Kanada; 3 Division of Gynecologic Oncology, Department of Obstetrics and Gynecology, University of British Columbia, Vancouver, Kanada

**Keywords:** LAM, PECom, Extrapulmonale Manifestation, TSC, Retroperitoneum, LAM, PEComa, Extrapulmonal manifestation, TSC, Retroperitoneum

## Abstract

**Hintergrund:**

Die Lymphangioleiomyomatose (LAM) ist eine seltene progrediente low-grade Neoplasie, welche überwiegend bei jungen Frauen auftritt. Die Erkrankung ist primär für ihre pulmonale Manifestation mit zystischer Parenchymdestruktion bekannt, jedoch ist auch eine extrapulmonale Beteiligung möglich. LAM ist assoziiert mit Mutationen in den Tuberöse-Sklerose-Komplex(TSC)-Genen *TSC1* und *TSC2* und kann sporadisch oder im Kontext der hereditären Erkrankung TSC auftreten.

**Ziel:**

Sensibilisierung für extrapulmonale LAM-Befunde im Retroperitoneum und Beckenraum.

**Methodik:**

Es wurde eine Datenbankrecherche zu LAM-Befunden in gynäkologischen Operationspräparaten durchgeführt. HE-Schnittpräparate wurden reevaluiert und immunhistochemische Präparate begutachtet. Klinische Daten wurden hinsichtlich des Vorliegens einer pulmonalen LAM oder TSC ausgewertet.

**Ergebnisse:**

Insgesamt wurden 13 Fälle identifiziert. Das Alter der Patientinnen reichte von 32 bis 77 Jahren, wobei 8/13 Frauen ≤ 55 Jahre alt waren, 2 Patientinnen wiesen eine Anamnese von pulmonaler LAM und TSC auf. Die meisten Frauen wurden aufgrund gynäkologischer Malignome operiert. Die histologische Begutachtung zeigte in 10/13 Fällen LAM-Läsionen in 1 bis 9 Lymphknoten mit einer Größe von 0,5–12,0 mm, 3/13 Fälle wiesen eine extranodale LAM-Beteiligung im Retroperitoneum, Myometrium und Ovarhilus auf. Die Läsionen exprimierten überwiegend HMB45, Desmin und SMA.

**Diskussion:**

LAM ist eine seltene Erkrankung mit vorrangiger Lungenbeteiligung, jedoch ist auch eine extrapulmonale Manifestation möglich. Es ist entscheidend, LAM-Foci zu erkennen und zu berichten, um einen ersten Hinweis für eine bis dato nichtdiagnostizierte pulmonale LAM und/oder eine TSC zu geben.

## Abstract (en)

**Background:**

Lymphangioleiomyomatosis (LAM) is a rare, slow progressing, low-grade neoplasia that primarily effects young women. The disease is well known for its pulmonary involvement with cystic destruction, but extra-pulmonary disease may occur. LAM is associated with mutations in the *TSC 1* or *TSC 2* genes and may develop sporadically or in the context of hereditary disease tuberous sclerosis complex (TSC). Incident LAM may represent the sentinel finding of the disease.

**Objective:**

Raising awareness for rare extrapulmonary LAM lesions in retroperitoneum and pelvic cavity.

**Methods:**

Data-based research was performed for LAM in gynecological surgical specimens. H&E-stained slides were re-examined, and immunohistochemical stains were re-evaluated. Clinical data were retrieved for the presence pulmonary LAM or TSC.

**Results:**

A total of 13 cases were identified. The age of the patients ranged from 32 to 77 years, and 8/13 were ≤ 55 years. Two women had a history of pulmonary LAM and TSC. Most women underwent surgery for gynecological malignancy. On histological examination, 10/13 patients presented LAM in 1 to 9 lymph nodes with a lesion size of 0.5 to 12.0 mm, mainly located subcapsular or in the nodal parenchyma. Three of the 13 women showed extranodal involvement of the retroperitoneum, myometrium, and the hilum of the ovary. Immunohistochemically LAM was positive for HMB45, desmin, and smooth muscle actin.

**Conclusion:**

LAM is a rare systemic disease that mainly involves the lungs. Nevertheless, extra-pulmonary manifestations may occur. It is important to report the incidental finding of even small foci of LAM within the pathology report. Incidental LAM may represent the sentinel lesion for pulmonary LAM and/or TSC.

Die Lymphangioleiomyomatose (LAM) ist primär als pulmonale Erkrankung bekannt. Das Wissen um seltene extrapulmonale Manifestationen ist eher gering. Mit diesem Beitrag soll anhand einer Fallserie von 13 inzidentell diagnostizierten LAM-Fällen in gynäkologischen Operationspräparaten Augenmerk auf die extrapulmonale LAM und ihre konsekutiven klinischen Implikationen gerichtet werden.

Die Lymphangioleiomyomatose (LAM) ist eine seltene, langsam progrediente low-grade Neoplasie, welche überwiegend bei jungen, prämenopausalen Frauen auftritt [14]. Weltweit wird eine Prävalenz von 3,4–7,8/1.000.000 Frauen mit LAM geschätzt, wobei von einer großen Zahl nichtdiagnostizierter Fälle ausgegangen wird [6]. Die LAM ist assoziiert mit Mutationen in den beiden Tuberöse-Sklerose-Komplex-Genen *TSC1* und *TSC2*. Sie kann sporadisch auftreten durch somatische Mutation vor allem im *TSC2*-Gen mehrheitlich bei Frauen (sog. sporadische LAM) oder im Zusammenhang mit der hereditären Erkrankung Tuberöse Sklerose Komplex aufgrund von Keimbahnmutationen in den Genen *TSC1* oder *TSC2* bei beiden Geschlechtern (sog. TSC-LAM) [3, 14]. Die genannten Gene codieren hierbei die Tumorsuppressorproteine Hamartin (*TSC1*-Gen) und Tuberin (*TSC2*-Gen), welche zusammen ein Dimer bilden und über weitere Signalproteine eine Aktivierung des mTORC1-Komplexes, einer wichtigen zellulären Signalschaltstelle, verhindern. Der mTORC1-Komplex besteht aus den Untereinheiten mTOR, Raptor und LST8, wobei insbesondere mTOR als pharmakologische Zielstruktur fungieren kann [10].

Üblicherweise steht im Vordergrund der LAM eine pulmonale Erkrankung mit einem diffusen dünnwandigen, zystischen Umbau und Destruktion des Lungenparenchyms mit daraus resultierender abnehmender Lungenfunktion mit Dyspnoe sowie rekurrentem Spontanpneumothorax [3]. Eher selten werden extrapulmonale Befunde der LAM erhoben, beispielsweise als LAM-Herde im Mediastinum, Retroperitoneum und intrapelvin [7, 13, 18, 22]. Trotz benigner Morphologie zeigt die Erkrankung Potenzial zum Rezidiv und zur Metastasierung bei Nachweis von LAM-Zellen in Blut- und Lymphgefäßen. Das Ursprungsorgan bzw. die Ursprungszelle der LAM ist bisher unbekannt, wobei unter anderem ein Primum im Uterus bzw. in der Lunge in der Fachliteratur diskutiert werden [4, 11].

## Histopathologische Charakterisierung

Entsprechend der aktuellen WHO-Klassifikation der thorakalen und uterinen Tumoren [24, 25] wird die LAM zugehörig zur Familie der perivaskulär epitheloidzelligen Tumoren (engl. „PEComatous tumours“) geführt. Zur Familie der PECome zählen hierbei auch die Angiomyolipome der Niere sowie klarzellige myomelanozytische Tumoren verschiedener Lokalisationen, wobei letztgenannte Entität in der Lunge als „sugar tumor“ („clear cell tumor of the lung“, CCTL) bezeichnet wird [25]. Histomorphologisch zeigt die LAM sich als überwiegend spindelzellige, gering epitheloide Zellpopulation (sog. LAM-Zellen) mit spärlich Mitosen und blandem Erscheinungsbild, partiell durchzogen von spaltartigen Hohlräumen [7, 14]. Immunhistochemisch exprimieren die Zellen glattmuskuläre (v. a. SMA, Desmin) und melanozytäre Marker (v. a. HMB45) sowie den Östrogen- und Progesteronrezeptor [8, 9, 14].

Anhand der vorliegenden Fallserie sind Befunde der äußerst selten diagnostizierten extrapulmonalen LAM im gynäkologischen Operationsgut zusammengestellt.

## Methodik

Es wurde eine Datenbankrecherche am Vancouver General Hospital (Kanada) zu LAM-Befunden in gynäkologischen Operationspräparaten durchgeführt. Hämatoxylin-Eosin-gefärbte und immunhistochemisch gefärbte Schnittpräparate wurden erneut herausgesucht und reevaluiert bezüglich Anzahl, Lokalisation, Größe und Immunphänotyp der LAM-Läsionen.

Weiterhin wurden klinische Daten zum Patientinnenalter bei Operationszeitpunkt, Vorliegen einer pulmonalen LAM-Manifestation, TSC sowie Art und Hauptindikation des Operationseingriffes zusammengetragen.

## Ergebnisse

### Klinische Daten

Es konnten 13 Fälle mit Nachweis von extrapulmonaler LAM in gynäkologischen Operationspräparaten zusammengestellt werden (Tab. 1). Die Altersspanne der Patientinnen reichte von 32 bis 77 Jahren mit einem Mittelwert von circa 52 Jahren; 8/13 der Frauen waren hiervon ≤ 55 Jahre alt. Bei einer Patientin waren klinisch ein TSC und bei einer anderen Patientin eine Manifestation der LAM im Bereich von Lunge, Abdomen und Becken bekannt gewesen. Insgesamt wurden 9/13 Frauen primär aufgrund unterschiedlicher gynäkologischer Malignome operiert, 2/13 Frauen aufgrund einer Endometriose und atypischer Endometriumhyperplasie sowie 2/13 Frauen aufgrund eines symptomatischen retroperitonealen Tumors bzw. pelviner Lymphadenopathie.

**Tab. 1:** Klinisch-pathologische Daten der Fallserie zur Lymphangioleiomyomatose (*n* = 13)

Fall	Alter (Jahre)	TSC/LAM (klinisch bekannt)	Art des Operationseingriffes	Lymphangioleiomyomatose			Primäre Operationsindikation
				Lokalisation	Größe	Uni-/multifokal	
1	32	Nein	HE mit Tumorresektion, AE bds., OE und LNE	1/2 pelvine LK (subkapsulär, extranodal)	1,1 mm	Unifokal	Low-grade endometriales Stromasarkom
2	34	TSC	LK-Biopsie	1/1 pelviner LK (intraparenchymal)	2,7 mm	Multifokal	Pelvine Lymphadenopathie
3	39	Nein	AE rechts, LNE, OE und Peritoneal-Biopsien	1/10 pelvine LK (subkapsulär, intraparenchymal, extranodal)	5,5 mm	Unifokal	Endometrioides Adenokarzinom des Ovars
4	52	Nein	HE mit AE bds., OE und LNE	4/16 pelvine und paraaortale LK (subkapsulär, intraparenchymal, extranodal)	1,1 bis 3,3 mm	Multifokal	Klarzelliges Karzinom des Ovars
5	53	Nein	HE mit AE bds. und SLNE	1/3 pelvine LK (subkapsulär, extranodal)	1,2 mm	Unifokal	Gemischtes Endometriumkarzinom
6	55	Nein	HE mit AE bds. und LNE	3/5 pelvine LK (subkapsulär, intraparenchymal, extranodal)	5,5 bis 7,7 mm	Unifokal	Adenokarzinom der Cervix uteri
7	58	Nein	HE mit AE bds. und SLNE	3/7 pelvine LK (subkapsulär, intraparenchymal)	0,5 bis 2,3 mm	Unifokal	Endometrioides Adenokarzinom des Endometriums
8	66	Nein	HE mit AE bds., OE und SLNE	1/3 pelvine LK (subkapsulär)	1,1 mm	Unifokal	Karzinosarkom des Endometriums
9	67	Nein	HE mit LNE	9/12 pelvine LK (subkapsulär, intraparenchymal)	1,0 bis 12,0 mm	Multifokal	Karzinosarkom des Endometriums
10	77	Nein	HE mit AE bds. und LNE	2/10 pelvine LK (subkapsulär, intraparenchymal, extranodal)	1,9 bis 11,0 mm	Unifokal	Endometrioides Adenokarzinom des Endometriums
11	33	Keine Daten verfügbar	HE mit pelvinen Biopsien	Myometrium (äußere Hälfte)	2,2 mm	Unifokal	Endometriose
12	41	LAM (Lunge, Abdomen, Becken)	AE bds. mit Tumorresektion	Retroperitoneum	27,0 mm	Unifokal	Symptomatischer retroperitonealer Tumor
13	65	Nein	HE mit AE bds.	Ovarhilus	4,9 mm	Unifokal	Atypische Endometriumhyperplasie

*HE* Hysterektomie, *AE bds.* Adnexektomie beidseits, *OE* Omentektomie, (*S)LNE* (Sentinel‑) Lymphonodektomie, *LK* Lymphknoten, *LAM* Lymphangioleiomyomatose, *TSC* Tuberöse Sklerose Komplex

### Histomorphologische und immunhistochemische Analyse

**Abb. 1:**
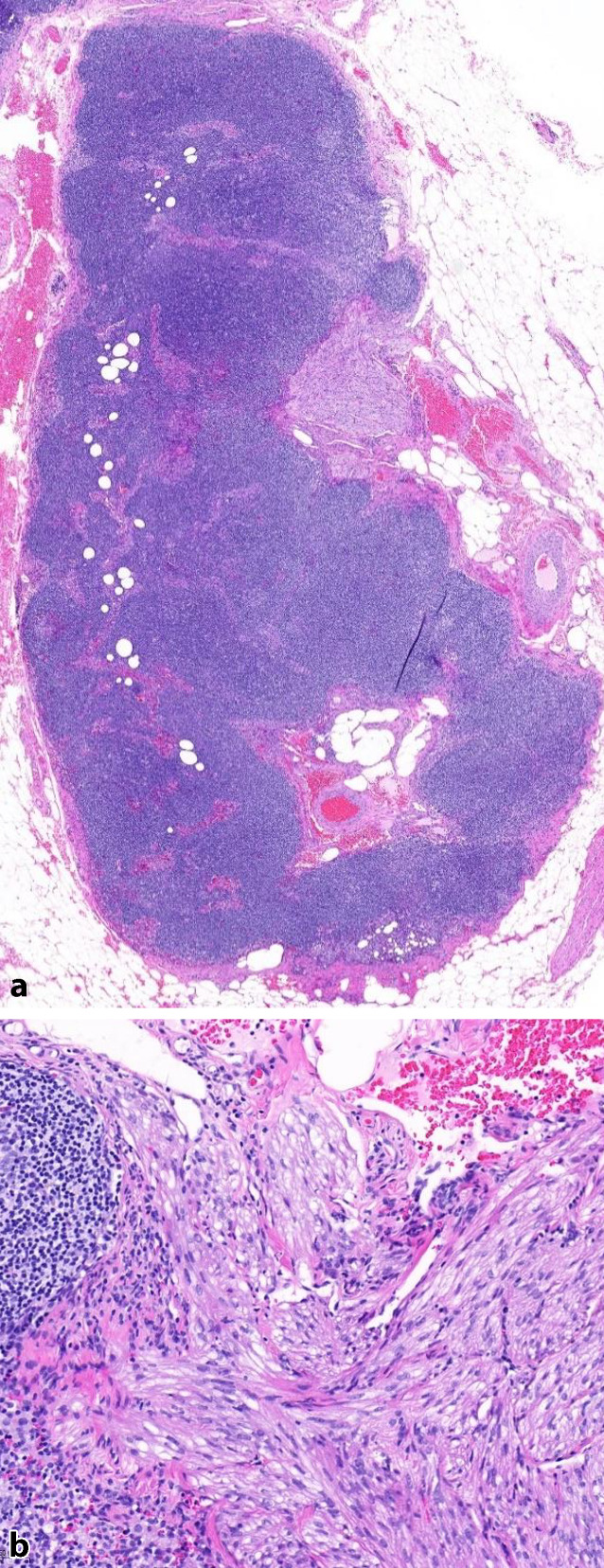
**a**,**b** Subkapsuläre Lymphangioleiomyomatose(LAM)-Infiltrate im Lymphknoten

**Abb. 2:**
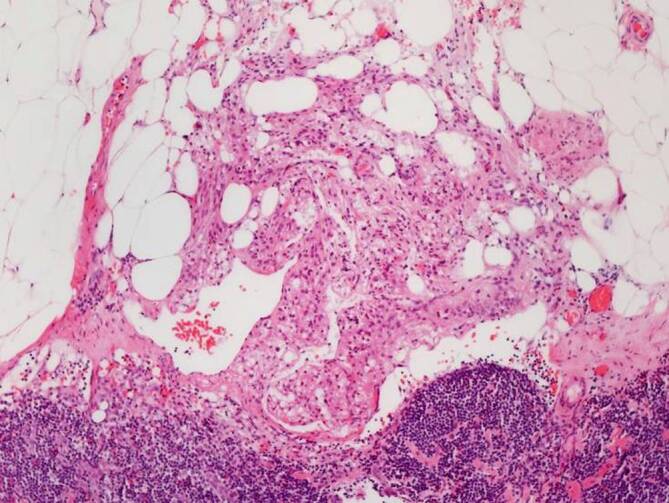
Extranodale Ausbreitung von Lymphangioleiomyomatose(LAM)-Infiltraten in das angrenzende Fettgewebe

**Abb. 3:**
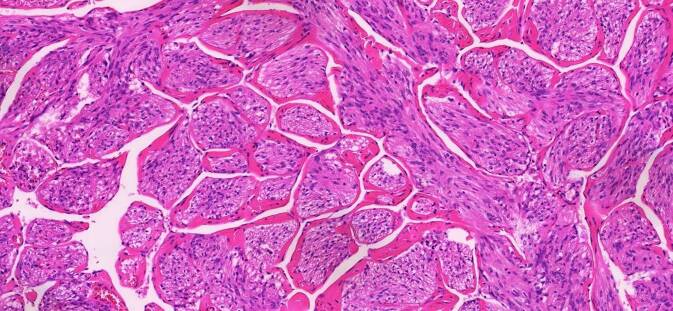
Lymphangioleiomyomatose(LAM)-Morphologie: blande, überwiegend spindelige Zellproliferate durchzogen von spaltartigen Hohlräumen

**Abb. 4:**
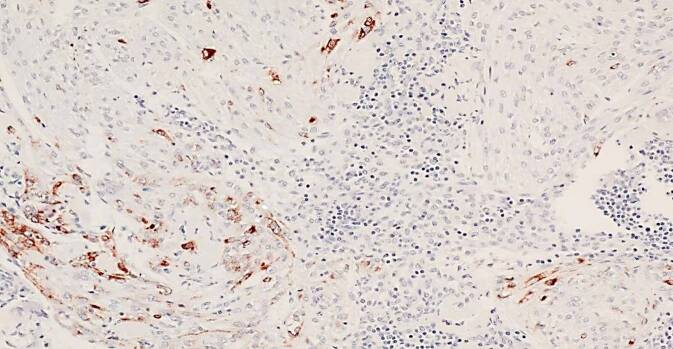
Immunhistochemisch fleckförmige Expression von HMB45 in Lymphangioleiomyomatose(LAM)-Zellen

In 10/13 Fällen zeigte sich eine LAM in 1 bis 9 pelvinen Lymphknoten. Die LAM-Läsionen präsentierten sich überwiegend unifokal subkapsulär und intraparenchymal im Lymphknoten (Abb. 1a, b), vereinzelt auch extranodal im angrenzenden Weichgewebe (Abb. 2). Die nodale LAM wies eine Größe von 0,5–12,0 mm auf. In 3/13 Fällen wurde LAM im Myometrium, Ovarhilus und Retroperitoneum detektiert mit einer Größe von 2,2–27,0 mm. Neben der typischen spindelzelligen LAM-Morphologie (Abb. 3) zeigte die LAM immunhistochemisch eine Expression des melanozytären Markers HMB45 (Abb. 4, fokal/mosaikartig 10/13) sowie der glattmuskulären Marker Desmin (4/7) und SMA (3/3), weiterhin eine Negativität gegenüber Breispektrum-Zytokeratinen (6/6) und S100 (3/3).

## Diskussion

Die LAM ist eine seltene progrediente Erkrankung mit Hauptmanifestation im Bereich der Lunge mit einer medianen Überlebenszeit ohne Lungentransplantation von etwa 20 Jahren [5]. Im Rahmen der Erkrankung können auch extrapulmonale LAM-Herde auftreten. Bisher gibt es in der Literatur nur wenige Fallberichte von LAM im Bereich des Retroperitoneums und des weiblichen Genitaltraktes mit nur einzelnen größeren Fallserien. Rabban et al. [19] dokumentieren 26 Fälle von inzidentell diagnostizierter LAM in pelvinen und paraaortalen Lymphknoten, Schoolmester et al. berichten 19 Fälle ebenfalls inzidentell entdeckter LAM in pelvinen und retroperitonealen Lymphknoten [20], Matsui et al. [13] 22 Fälle von extrapulmonaler LAM in Lymphknoten von Mediastinum und Retroperitoneum sowie Hayashi et al. [7] 10 Fälle von LAM in Uterus und Adnexen. Die Fallserie von Rabban et al. [19] demonstriert klinisch okkulte LAM in Lymphknoten, welche nur geringe Ausmaße von 1–19 mm aufweisen mit einem Mittelwert von 3,5 mm Größe. Die Forschungsgruppe zeigt hierbei eine eher seltene Assoziation von extrapulmonaler LAM mit pulmonaler LAM bzw. TSC. Schoolmester et al. zeigen ähnliche Ergebnisse mit singulärer oder multipler nodaler LAM von 1–9 mm bei einem Mittelwert von 4,3 mm. Die Forschenden untersuchten zudem Daten aus Verlaufskontrollen über 3–123 Monaten und konnten hierbei keine zusätzliche Entwicklung einer pulmonalen LAM dokumentieren [20]. Im Gegensatz dazu schildern Matsui et al. [13] extrapulmonale LAM-Lymphknoten-Manifestationen mit konsekutiv im Verlauf von 1–2 Jahren diagnostizierter pulmonaler LAM in einer Mehrzahl ihrer Fälle. Hayashi et al. [7] weisen darauf hin, dass die untersuchte Studienpopulation mit vorbekannter pulmonaler LAM zu 90 % LAM-Läsionen im Uterus aufzeigen und dass alle uterinen LAM-Läsionen begleitet waren von LAM-Herden in retroperitonealen und pelvinen Lymphknoten. Ähnlich zu diesen Ergebnissen finden sich in der Literatur wiederkehrend Fallberichte bzw. Serien einzelner Fälle mit Detektion von uteriner und/oder nodaler LAM bei Patienten mit pulmonaler LAM oder TSC [1, 12, 15, 18].

Unsere Daten stehen überwiegend in Einklang mit denen von Rabban et al. [19] und Schoolmester et al. [20]. Inzidentelle LAM-Läsionen konnten in den uns vorliegenden Fällen überwiegend als mikroskopisch kleine Herde in Lymphknoten nachgewiesen werden. Eine Assoziation mit TSC oder pulmonaler LAM war selten (Tab. 1). Inwieweit inzidentell diagnostizierte nodale LAM hierbei mit pulmonaler LAM und TSC in Beziehung steht, wird in der Literatur kontrovers angegeben und bleibt unklar [19, 27].

### Klinische Implikationen

Mehrheitlicher Konsensus ist, dass eine inzidentell diagnostizierte extrapulmonale LAM weitergehende Abklärung hinsichtlich des Vorliegens einer pulmonalen LAM bzw. der hereditären Erkrankung TSC bedarf [15, 26]. Da die pulmonale LAM zu einer verminderten Lebenserwartung mit ggf. Notwendigkeit einer Lungentransplantation aufgrund von progredienter Lungenfunktionseinschränkung führen kann [3, 14], ist eine frühzeitige Diagnose für die Prognose der Patientinnen entscheidend. Ebenfalls ist das Wissen um eine TSC-Erkrankung wichtig, um anderweitige assoziierte Tumorerkrankungen zeitnah zu diagnostizieren [15]. Als zielgerichtete medikamentöse Therapie werden zurzeit die mTOR-Inhibitoren Sirolimus und Everolimus geführt [2, 23], wobei derzeit lediglich für Sirolimus eine Zulassung bei LAM existiert [16, 17]. Die genannten Medikamente verhindern den Progress, bewirken jedoch keine Heilung der Erkrankung [9, 11]. Zur weiteren Charakterisierung der Erkrankung und Erforschung neuer medikamentöser Therapieoptionen bedarf es daher fortlaufender Sensibilisierung für die LAM [11].

### Histopathologische Differentialdiagnosen

In der Histopathologie sind von der LAM differentialdiagnostisch vor allem glattmuskuläre Neoplasien zu unterscheiden, unter anderem von der nodalen LAM intranodale Leiomyome und Lymphknotenbeteiligung bei diffuser Leiomyomatose oder disseminierter peritonealer Leiomyomatose. Zudem ist von der uterinen LAM das endometriale Stromasarkom abzugrenzen [7, 19]. Schlüssel zur LAM-Diagnose ist hierbei in erster Linie die Histomorphologie als mikroskopisch kleine, teils multifokale, blande, spindelige Läsion durchzogen von spaltartigen Hohlräumen [7]. Immunhistochemisch kann die Diagnose durch melanozytäre und glattmuskuläre Marker, Hormonrezeptoren und D2–40 untermauert werden. Wegweisend ist insbesondere HMB45 als melanozytärer Marker mit in der LAM üblicherweise fokaler, schwacher Expression. In der Literatur wird in Einzelfällen auch Negativität gegenüber HMB45 berichtet [1, 19], sodass alternativ weitere melanozytäre Marker wie MiTF oder Melan‑A herangezogen werden können [19]. In einer kürzlich veröffentlichten Publikation stellte sich in einer kleinen LAM-Kohorte auch Positivität gegenüber PRAME dar [21]. Weiterhin zeigt die LAM typischerweise eine kräftige diffuse Reaktivität gegenüber glattmuskulären Markern (SMA, Desmin) sowie Positivität der Hormonrezeptoren (Östrogen- und Progesteronrezeptor) [13, 19], wobei beide Markergruppen wenig hilfreich zur Differenzierung glattmuskulärer Neoplasien sind. Zusätzlich lassen sich mittels D2–40 die teils unscheinbaren Lymphgefäßkanälchen innerhalb der LAM darstellen [19]. Darüber hinaus in Abgrenzung der LAM zum klassischen PECom weist letztgenannte Entität üblicherweise eine größere, unifokale Tumormasse auf mit überwiegend epitheloiden Tumorzellen, welche in einem Teil der Fälle mit einer *TFE3*-Mutation assoziiert sein kann [25].

In Anlehnung zu unseren Daten zeigten sich überwiegend mikroskopisch kleine LAM-Herde mit entsprechender Morphologie und immunhistochemischem Expressionsmuster. Lediglich ein retroperitonealer Befund imponierte als 2,7 cm großer Tumor und wurde insbesondere aufgrund vorbekannter pulmonaler und abdominaler LAM der Patientin als weiterer LAM-Herd eingeordnet.

Abschließend ist es entscheidend, die LAM als Zufallsbefund histopathologisch anhand der typischen LAM-Morphologie mit entsprechendem immunhistochemischem Profil (melanozytäre und glattmuskuläre Marker, Hormonrezeptoren, D2–40) zu diagnostizieren und auf eine klinische, ggf. humangenetische Abklärung bezüglich einer hereditären Erkrankung (TSC-Syndrom) hinzuweisen [9, 14].

## Fazit für die Praxis

- Die Lymphangioleiomyomatose (LAM) ist eine seltene, langsam progrediente low-grade Neoplasie überwiegend jüngerer Frauen, welche sowohl im Bereich der Lungen als auch extrapulmonal auftreten kann.
- Es wird zwischen 2 Subtypen der LAM unterschieden: sporadische LAM versus TSC-LAM (LAM assoziiert mit Tuberöse Sklerose Komplex).
- Extrapulmonale LAM-Läsionen zeigen sich vor allem im Mediastinum, Retroperitoneum oder intrapelvin inklusive Lymphknoten und weiblichem Genitaltrakt.
- Es ist entscheidend, extrapulmonale LAM histopathologisch zu diagnostizieren für eine frühzeitige klinische Abklärung hinsichtlich einer pulmonalen LAM bzw. eines TSC-Syndroms.

## Data Availability

Die erhobenen Datensätze können auf begründete Anfrage in anonymisierter Form beim korrespondierenden Autor angefordert werden.
